# Safety profile of sodium glucose co-transporter 2 (SGLT2) inhibitors: A brief summary

**DOI:** 10.3389/fcvm.2022.1010693

**Published:** 2022-09-21

**Authors:** Annamaria Mascolo, Raffaella Di Napoli, Nunzia Balzano, Donato Cappetta, Konrad Urbanek, Antonella De Angelis, Lucia Scisciola, Irene Di Meo, Maria Giuseppa Sullo, Concetta Rafaniello, Liberata Sportiello

**Affiliations:** ^1^Campania Regional Centre for Pharmacovigilance and Pharmacoepidemiology, Naples, Italy; ^2^Department of Experimental Medicine – Section of Pharmacology “L. Donatelli”, University of Campania “Luigi Vanvitelli”, Naples, Italy; ^3^Department of Molecular Medicine and Medical Biotechnologies, University of Naples Federico II, Naples, Italy; ^4^CEINGE-Biotecnologie Avanzate, Naples, Italy; ^5^Department of Advanced Medical and Surgical Sciences, University of Campania “Luigi Vanvitelli”, Naples, Italy

**Keywords:** safety, SGLT2 (sodium-glucose cotransporter 2) inhibitor, adverse drug (event), evidence medicine, review

## Abstract

A new therapeutic class of oral agents firstly used for the treatment of type 2 diabetes mellitus is represented by gliflozines or sodium-glucose co-transporter 2 (SGLT2) inhibitors. SGLT2 inhibitors might be effective alone or in combination with any other drugs. This therapeutic class currently includes five agents: canagliflozin, dapagliflozin, empagliflozin, ertugliflozin, and sotagliflozin. SGLT2 inhibitors prevent the renal reabsorption of filtered glucose and sodium by blocking the SGLT2 co-transporters in the proximal convoluted renal tubule, facilitating glucose excretion in the urine (glycosuria) and lowering blood glucose levels. SGLT2 inhibitors have also shown to have pleiotropic effects and determine cardiovascular and renal prevention, thus leading to an extension of their therapeutic indication to include the heart failure. Despite their clinical benefits, warnings about adverse events have been implemented by Regulatory Agencies in the product's information since their introduction to the market. In particular, SGLT2 inhibitors have shown a strong impact on a high number of risk factors. They can cause hypoglycaemia, hypotension, lower limb amputation, fractures, genito-urinary infections, and diabetic ketoacidosis with different frequencies of onset. Despite some of these events are rare, they can lead to serious and dangerous complications, highlighting the importance of a strict monitoring of patients. Overall, SLGT-2 inhibitors are effective antidiabetic drugs with favorable advantages in renal and cardiovascular protection, and with a generally well-tolerated safety profile. This review aims to summarize the safety profile of SGLT2 inhibitors available in the market.

## Introduction

Gliflozines or Sodium Glucose Co-Transporter 2 (SGLT2) inhibitors are a class of drugs firstly introduced for the treatment of type 2 diabetes mellitus (1). The name of this drug class comes from their pharmacological target “the SGLT2.” The SGLT is a membrane protein capable of co-transporting sodium ions (Na+) and glucose into cells. In humans, we have 6 types of SGLT, those of type I and II are most responsible for the absorption of glucose. In particular, SGLT1 is located mainly at the level of the intestinal mucosa and also allows the absorption of galactose, while SGLT2 is mainly present in the proximal tubule of the renal nephron and it is responsible for the 90% of renal glucose reabsorption (2).

The class of SGLT2 inhibitors includes five oral drugs: canagliflozin, dapagliflozin, empagliflozin, ertugliflozin, and sotagliflozin. Also other SGLT2 inhibitors have been developed, but are only approved in Japan. Initially, these drugs were developed to inhibit the absorption of glucose mediated by SGLT2 at the level of the proximal renal tubule, thus favoring glycosuria and managing hyperglycaemia. To date, such drugs are known to have pleiotropic affects and able to bring significant, metabolic, renal, and cardiovascular benefits, also allowing to reduce levels of glycated hemoglobin (HbA1c), blood pressure, and body weight (1). These drugs can be used in monotherapy or in combination with other hypoglycaemic agents. Therapeutic indications of this drug class include type 1 and 2 diabetes mellitus, heart failure, and chronic kidney disease. Pharmacological characteristics of SGLT2 inhibitors are shown in Table 1. Type 2 diabetes mellitus is a disease characterized by insulin resistance and decreased beta cell function of pancreas. Among the risk factors for the onset of this pathology, we find the genetic predisposition. Type 2 diabetes mellitus affects about 7% of the population worldwide and is expected to increase to 300 million patients by 2025 (3). Type 2 diabetes mellitus is a cardio-renal-metabolic condition, sometimes associated with other diseases, such as heart failure and atherosclerotic cardiovascular disease (ASCVD) (4). This pharmacological class was also associated with a consistent improvement of cardiovascular outcomes in patients with type 2 diabetes mellitus with concomitant cardiovascular or chronic kidney disease, and in patients with heart failure (5). However, despite their clinical advantages, SGLT2 inhibitors have also been associated with the occurrence of adverse events. This narrative review aims to summarize the safety profile of SGLT2 inhibitors, in particular by bringing out data on adverse events that most frequently occurred following their use.

**Table 1:** Pharmacological characteristics of SGLT2 inhibitors available in the market.

**SGLT2 inhibitors**	**Therapeutic indication**	**Dosing**	**Age**	**Type of therapy**
Canagliflozin	Type 2 diabetes mellitus	100 mg once a day	Adult patients	Monotherapy or association
Dapagliflozin	Type 2 diabetes mellitus	10 mg once a day	Adult patients; children from	Monotherapy or association
	Heart failure	10 mg once a day	10 years of age (only for type 2	
	Chronic kidney disease	10 mg once a day	diabetes mellitus)	
Empagliflozin	Type 2 diabetes mellitus	10 mg once a day	Adult patients	Monotherapy or association
	Heart failure	10 mg once a day		
Ertugliflozin	Type 2 diabetes mellitus	5 mg or 10 mg once a day	Adult patients	Monotherapy or association
Sotagliflozin	Type 1 diabetes mellitus	200 mg or 400 mg once a day	Adult patients	Monotherapy or association

## Mechanism of action of SGLT2 inhibitors

The SGLT2 inhibitors block the SGLT2 in a selective and potent way (6). SGLT2 are high-capacity, low-affinity transporters, present in the proximal convoluted renal tubule, and responsible for ~90% reabsorption of filtered plasma glucose. SGLT2 inhibition simply inhibits the transporter and prevents the renal reabsorption of filtered glucose and sodium, reducing hyperglycaemia and facilitating glucose excretion in urine (7). SGLT2 inhibitors also have a variable cross-reactivity with SGLT1, which is clinically important only for canagliflozin (IC50 for SGLT1: 684 nM; for SGLT2: 4.4 nM). Indeed, the other SGLT2 inhibitors have higher half maximal inhibitory concentration for SGLT1 than SGLT2 (dapagliflozin: 803 vs, 1.6; empagliflozin: 8,300 vs. 3.1; ertugliflozin: 1,960 vs. 0.9). Sotagliflozin instead acts similarly on SGLT1 and SGLT2, being considered as a dual SGLT inhibitor (8). The mechanism of action of SGLT2 inhibitors is independent of insulin sensitivity, for this reason these drugs represent a new therapeutic approach that takes action directly on the kidneys. There is no risk of overstimulation of the pancreatic beta cells and minimal risk of hypoglycaemia (9). Other potential effects of SGLT2 inhibition include reductions in albuminuria, weight loss and lipid metabolism shift, improvement in hemoglobin levels and reducing oxygen demand and cellular glucotoxicity. Moreover, SGLT2 inhibitors appear to reduce levels of inflammatory cytokines, such as IL-6, TNF, IFNγ, NF-κβ, TLR-4, and TGF-β, and improve mitochondrial function (10).

## Safety of SGLT2 inhibitors

Several adverse events have been observed during early clinical trials with SGLT2 inhibitors. A typical adverse event with glucose lowering agents is the hypoglycaemia. A recent meta-analysis has not shown an increased risk of hypoglycaemia with the administration of SGLT2 inhibitors alone, and this is in accordance with the mechanism of action of these drugs that are not able to increase the release of insulin or influence the glucose synthesis (11). However, this risk can increase if SGLT2 inhibitors are associated with other glucose-lowering agents, including insulin. Not all adverse events initially observed with SGLT2 inhibitors have been confirmed by other clinical trials. This is the case of urinary tract infections (UTIs) that in a meta-analysis showed no association with the use of SGLT2 inhibitors (Risk Ratio, RR: 0.97; 95% CI: 0.81–1.16) (12). In this regards, it should be also considered that the diabetes itself can predispose to UTIs development (13), since high urinary glucose levels can facilitate the growth of commensal microorganisms in the urinary tract (13). Despite the risk of UTIs is not increased by SGLT2 inhibitors, the risk of genital infections seems higher with this drug class. Indeed, the rate of this events was higher with empagliflozin than placebo in the EMPA-REG clinical trial (14). Accordingly, two meta-analyses showed a risk of 3.3 (95% CI, 2.74–3.99) (1, 15) and 2.86 (95% CI, 2.00–4.10) (12) for genital infection with the use of SGLT2 inhibitor in type 2 diabetes mellitus patients. Moreover, a retrospective cohort study found an adjusted hazard ratio (HR) for SGLT2 inhibitors vs. DPP-4 inhibitors of 2.81 (95% CI, 2.64–2.99) for women, and of 2.68 (95% CI, 2.31–3.11) for men. Similar findings were observed in the comparison between SGLT2 inhibitors and GLP-1 agonists, with higher hazards identified in patients aged ≥60 years (HR, 4.45; 95% CI, 3.83–5.17 in women and HR, 3.30; 95% CI, 2.56–4.25 in men), and no significant difference observed across single SGLT2 inhibitors (16). Another study, investigating the incidence rate ratio (IRR) of urogenital infections during treatment with SGLT2 inhibitors compared with a non-exposure period, found an increased risk for UTIs (IRR 1.25, 95% CI 1.14–1.37) and genital infections (IRR 1.44, 95% CI 1.28–1.62) in women aged ≥50 years. The highest risk was observed 8–14 and 15–28 days after initiating the SGLT2 inhibitor for UTI (IRR 1.49, 95% CI 1.07–2.08) and genital infections (IRR 2.11, 95% CI 1.66–2.67), respectively (17).

A serious type of genital infection is the Fournier's gangrene, which is a rare but potentially fatal event characterized by necrotizing fasciitis of the perineal soft tissues (18). A recent evidence found an association between the administration of an SGLT2 inhibitor and the onset of Fournier's gangrene, with 55 cases identified over a period of 6 years (19). Regulatory Agencies such as US Food and Drug Administration (FDA) and European Medicine Agency (EMA) have updated the product's information of SGLT2 inhibitors to include a warning about the risk of this event (20). Diabetic ketoacidosis (DKA) is a rare but potentially fatal event that was found associated with the use of SGLT2 inhibitors (12). The risk of DKA was highlighted in clinical trials (21–24), leading the Pharmacovigilance Risk Assessment Committee of EMA to declare it for the entire class of SGLT-2 inhibitors (25). The hypothesized mechanism by which SGLT2 inhibitors may induce DKA include a reduction in insulin secretion due to the significant decrease in blood glucose level, with consequent increased synthesis of free fatty acids that are transformed into ketone bodies, and the increase in glucagon secretion which leads to ketone body synthesis (26). Specifically, the renal inhibition of SGLT2 induces glycosuria and a reduction of lipolysis with an increase in ketone reabsorption and circulating ketone levels. Moreover, as a result of glycosuria and through a direct action on pancreatic α-cells, SGLT2 inhibitors increase the release of glucagon from pancreas, which induces an increase in lipolysis and ketogenesis in the liver (27, 28). The risk of DKA was found higher in patients with type 1 diabetes mellitus and has led the withdrawal of this therapeutic indication for dapagliflozin (29). The increased frequency of DKA observed in this subpopulation can be explained by the renal and pancreatic effects of SGLT2-inhibitors that are emphasized in patients in therapy with insulin, since to minimize the risk of hypoglycaemia it is necessary to further decrease the insulin dose. This can furtherly alter the glucagon/insulin ratio determining an increase in ketone body levels (30). In this regards, a previous pharmacovigilance study showed a higher reporting frequency of ketoacidosis with dapagliflozin when compared to Dipeptidyl peptidase-4 inhibitors or insulin (31). However, since dapagliflozin is still authorized for type 2 diabetes mellitus and the risk of ketoacidosis was observed with all SGLT2 inhibitors, patients treated with these drugs should be carefully monitored, also to reduce the dangerous complications of ketoacidosis. Patients treated with SGLT2 inhibitors who develop this event need to withdraw immediately the drug, evaluate ketone levels, and start insulin therapy (32).

Another event observed with this drug class is the onset of hypotension, which is strictly related to the mechanism of action of SGLT2 inhibitors and to volume depletion. A meta-analysis found indeed a significant reduction of systolic and diastolic blood pressures with SGLT2 inhibitors (12). However, this event can be beneficial in some patients with type 2 diabetes mellitus and potentially responsible of the cardiovascular protection of this drug class. Indeed, SGLT2 inhibitors have shown to reduce the risk of cardiovascular outcomes and mortality, and real-life data support contemporary society recommendations to prioritize their use in patients with diabetes mellitus and at high risk for cardiovascular complications (33). Cardiovascular benefits are related to the pleiotropic effects of SGLT2 inhibitors, which also include osmotic diuresis and natriuresis, reduction of body weight and visceral adiposity, decrease in uric acid, oxidative stress, and inflammation (34–37). Moreover, SGLT2 inhibitors do not increase the neurohormonal activation (RAAS and sympathetic pathways), leading to a positive left ventricular remodeling (49, 50) and highlighting a positive pharmacodynamic interaction between SGLT2 inhibitors and RAAS inhibitors (38). SGLT2 inhibitors can also directly target cardiomyocytes and endothelial cells by interacting with other channels and transporters present on cell surface, including the SGLT1, NHE1 and Nav1.5 (5). Another pleiotropic effect with a cardiac benefit is the reduction of NT-proBNP caused by SGLT2 inhibitors, which correlates with a reduction in ventricular pressure and distention, preload and both pulmonary and systemic congestion (5). Finally, SGLT2 inhibitors shift the fuel energetics of myocardium toward ketones, thus improving the myocardial work efficiency (37). Moreover, based on the results of two clinical trials (DAPA-HF and EMPEROR-Reduced), SGLT2 inhibitors are now recommended for the treatment of heart failure with reduced ejection fraction in association with standard treatments regardless of the presence of diabetes mellitus (5). Finally, based on the results from the EMPEROR-preserved trial, empagliflozin is also recommended in patients with a preserved ejection fraction (5). Benefit effects are also shown in patients with type 2 diabetes mellitus hospitalized for acute myocardial infarction and treated with SGLT2-inhibitors. Indeed, these patients showed a significant decline in inflammation and infarct size compared to non-SGLT2-inhibitors users. Interestingly enough, such results appeared to be independent of glucose-metabolic control. It should be highlighted such new open research area regarding the cardio-protective effects of SGLT2 inhibitors in the setting of coronary artery disease (39, 40). SGLT2 inhibitors may indeed influence the blood lipid profiles. Many studies have investigated their role in atherosclerosis (41–43), triglyceride levels (44), and non-alcoholic fatty liver disease (45, 46). A meta-analysis of 15 randomized trials (with a total of 7,578 patients with type 2 diabetes mellitus) found an increase in total cholesterol, low-density lipoprotein cholesterol, and high-density lipoprotein cholesterol, while a decrease in triglycerides with SGLT2 inhibitors compared to placebo or other oral glucose-lowering drugs. No risk of dyslipidemia was also identified with SGLT2 inhibitors (47).

Whether SGTL2 inhibitors increase the risk of amputation of lower limbs (toes, feet, or legs) was highly debated in the literature with clinical trials showing controversial results (12). In the CANVAS clinical trial program (48), canagliflozin had a higher risk of amputation than placebo. However, these results were not supported by the CREDENCE trial (49), which showed a similar incidence of amputation between canagliflozin and placebo, with absolute rates of 12.3 and 11.2 events/1,000 patient-years, respectively. CREDENCE trial results were supported by data from four administrative claims databases of United States (50). Although evidence on the risk of amputation are controversial, product's information have been updated to include a warning on this potential risk. Moreover, the American Diabetes Association (ADA) guidelines recommend to evaluate at least annually feet of patients with diabetes to identify risk factors for ulcers and amputations (1). Canagliflozin was also associated with the risk of bone fracture during the CANVAS program (51). However, this risk was not observed in other trials with canagliflozin, including the CREDENCE trial (49, 52). Moreover, other factors should be considered for the risk of fractures with SGLT2 inhibitors, including the predisposition of patients with type 2 diabetes mellitus (53), and the absence of a pathogenetic mechanism linking the onset of fractures with the use of SGLT2 inhibitors (54). A recent meta-analysis including data for canagliflozin, dapagliflozin, empagliflozin, and ertugliflozin showed no difference for the risk of fractures with these medicines compared to placebo (12).

**Figure 1:**
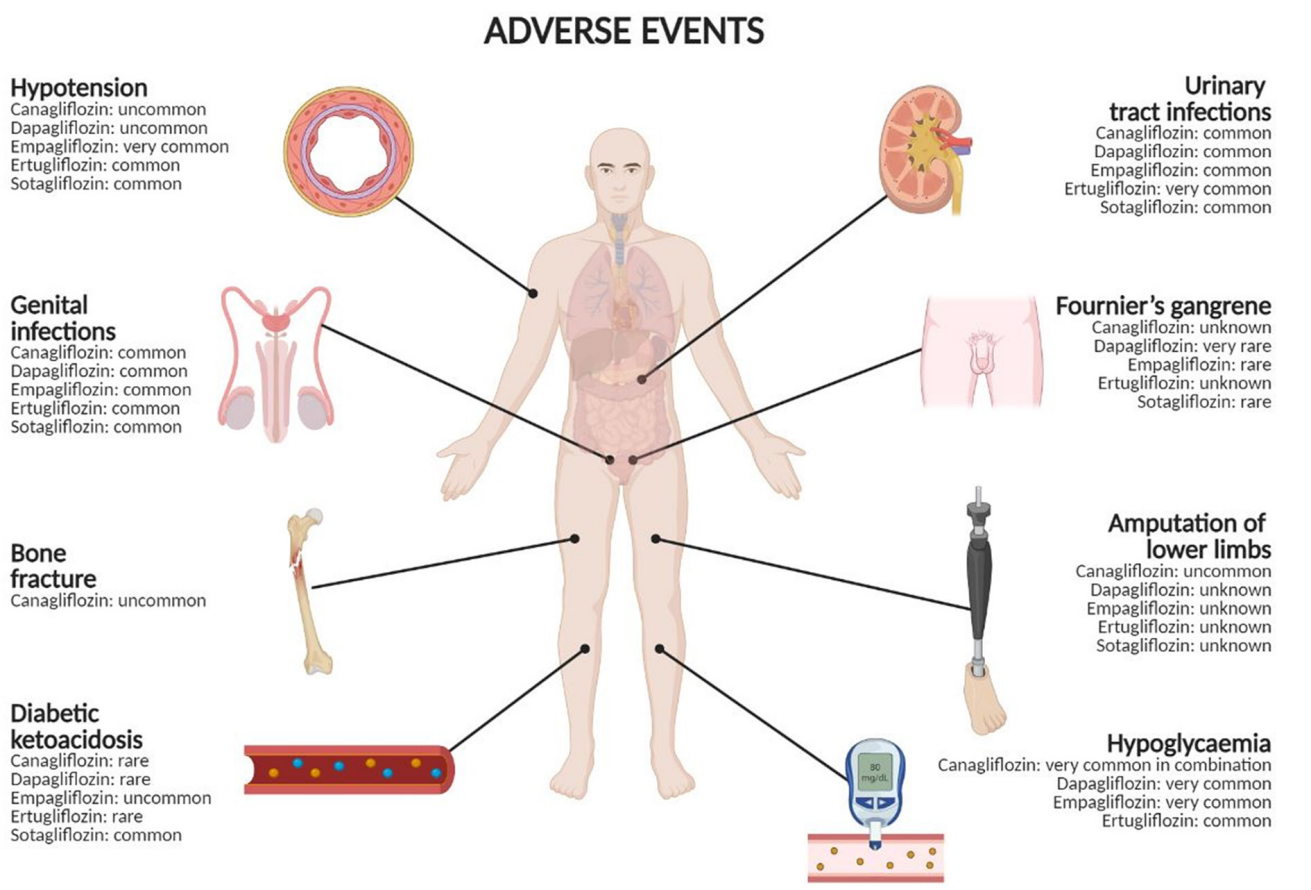
Adverse events with SGLT2 inhibitors and their frequencies. Frequency categories are defined according to the following convention: very common (≥1/10), common (≥1/100 to < 1/10), uncommon (≥1/1,000 to < 1/100), rare (≥1/10,000 to < 1/1,000), very rare (< 1/10,000), unknown (cannot be estimated from the available data). Created in BioRender.com.

Finally, in terms of safety, a recent case report showed myotoxicity secondary to the concomitant administration of rosuvastatin and canagliflozin (55). This patient had well-tolerated rosuvastatin for more than 5 years, but experienced severe muscle pain and hepatotoxicity after 15 days from initiating treatment with canagliflozin, with plasma rosuvastatin levels found higher than expected at the time of hospital admission. The authors speculated a pharmacokinetic interaction on drug transporters, with canagliflozin that led to an increase in intestinal absorption and a decrease of hepatocellular uptake and excretion of rosuvastatin. Authors highlight the potential for interaction between these drugs that should be considered in patients treated with both statins and SGLT2 inhibitors. Adverse events associated with SGLT2 inhibitors and their frequencies are reported in Figure 1 and characteristics of aforementioned safety clinical studies are shown in Table 2. Genital infections have a gender-dependent frequency of development that is higher for female than male. The role of different races/ethnicities on the safety profiles of SGLT2 inhibitors have been investigated. A meta-analysis showed a difference in the safety profiles of SGLT2 inhibitors between Asian and non-Asian patients with type 2 diabetes mellitus. Specifically, SGLT2 inhibitors were associated with an increased risk of UTIs compare to non-Asian patients, but the risk of UTI was similar to placebo in Asian patients (56). This meta-analysis also showed that the risk of hypoglycaemia was increased in non-Asian than Asian patients (56). Another meta-analysis found that SGLT2 inhibitors had similar risks of hypoglycaemia, urinary tract infection, genital infection, hypovolemia, and fracture compared to placebo in Japanese patients (57). Further evidence with longer follow-up and involving more ethnicities are needed to better clarify the role of races/ethnicities on the safety profiles of SGLT2 inhibitors. The use of SGLT2 inhibitors in the pediatric population is to date limited to dapagliflozin. Pediatric studies are few and still in an exploratory phase, making difficult the evaluation of the efficacy and safety of these drugs. Children have a hepatic and kidney function not fully developed; therefore, a more frequent and careful monitoring should be performed in pediatric patients with a special attention to genital infections, UTIs, hypotension, DKA, and hypoglycaemia. On the contrary, the risk of lower limb amputation seems to be lower in children with diabetes. However, the available evidence in pediatrics did not underline serious adverse events (58).

**Table 2:** Characteristics of safety clinical studies.

**References**	**Type of evidence**	**Study population**	**SGLT2 inhibitors**	**Comparison**	**Disease**	**Safety outcome considered**
Rosenstein and Hough (14)	RCT	7,020	Empagliflozin	Placebo	Type 2 diabetes	Genital infections
Dave et al. (16)	Cohort study	286,068	Canagliflozin, dapagliflozin or empagliflozin	DPP4 inhibitors (sitagliptin, saxagliptin, linagliptin or alogliptin)	Type 2 diabetes	Genital infections
Kang et al. (17)	Case-series	2,949	Dapagliflozin, empagliflozin, or ipragliflozin	Non-exposure period to SGLT2 inhibitor	Type 2 diabetes	UTIs and genital infections
Bersoff-Matcha et al. (19)	Case series	55	Cagliflozin, dapagliflozin, empagliflozin	–	Type 2 diabetes or not specified	Fournier's gangrene
Dandona et al. (21, 23)	RCT	833	Dapagliflozin	Placebo	Type 1 diabetes	Diabetic ketoacidosis
Phillip et al. (22)	Pooled study	1,646	Dapagliflozin	Placebo	Type 1 diabetes	Diabetic ketoacidosis
Mathieu et al. (24)	RCT	813	Dapagliflozin	Placebo	Type 1 diabetes	Diabetic ketoacidosis
Di Mauro et al. (31)	Pharmacovigilance study	2,406 cases with dapagliflozin	Dapagliflozin	DPP4 inhibitors or insulin	Type 1 and 2 diabetes	Diabetic ketoacidosis
Neal et al. (48, 51)	RCT	10,142	Canagliflozin	Placebo	Type 2 diabetes	Amputation, Bone fracture
Perkovic et al. (49)	RCT	4,401	Canagliflozin	Placebo	Type 2 diabetes	Amputation
Ryan et al. (50)	US administrative claims databases study	714,582	Canagliflozin, dapagliflozin, empagliflozin	non SGLT2 inhibitor	Type 2 diabetes	Amputation
Watts et al. (52)	RCT	10,194	Canagliflozin	Placebo	Type 2 diabetes	Bone fracture
Brailovski et al. (55)	Case report	1	Canagliflozin	–	Type 2 diabetes	Myotoxicity

Randomized Clinical Trial (RCT); Urinary tract infections (UTIs).

## Conclusion

The development of SGLT2 inhibitors has not only generated a new treatments option for diabetes mellitus, but also a strategy to prevent cardiovascular and renal complications, thus leading to an extension of the therapeutic indication for some molecules of this drug class. SGLT2 inhibitors are indeed the most promising strategy for heart failure with preserved ejection fraction, reaching a prominent position in the last European Society of Cardiology (ESC) guidelines on heart failure. The safety profile of SGLT2 inhibitors is generally good. Adverse events such infections, hypotension, amputation, fractures, and diabetic ketoacidosis have been reported in the Product's information by Regulatory Agencies and require a constant monitoring of patients. Indeed, despite most events are rare (ketoacidosis, amputations and Fournier gangrene), they can lead to serious and dangerous complications. An important aspect that may be enhanced in the future is the identification of predisposing or precipitating risk factors that could help to prevent the most severe complications. In conclusion, these adverse events do not alter the overall cardiovascular and renal benefits of SGLT2 inhibitors.

## Author contributions

AM, RDN, NB, DC, KU, ADA, LSc, IDM, MGS, CR, and LSp: drafting the work, revising it for important intellectual content, final approval of the version to be published, and agreement to be accountable for all aspects of the work in ensuring that questions related to the accuracy or integrity of any part of the work are appropriately discussed. CR and LSp: developed the concept and designed the study. AM and RDN: wrote the paper. All authors contributed to the article and approved the submitted version.

## Conflict of interest

The authors declare that the research was conducted in the absence of any commercial or financial relationships that could be construed as a potential conflict of interest.

## Publisher's note

All claims expressed in this article are solely those of the authors and do not necessarily represent those of their affiliated organizations, or those of the publisher, the editors and the reviewers. Any product that may be evaluated in this article, or claim that may be made by its manufacturer, is not guaranteed or endorsed by the publisher.
